# Hoagland's Sign as a Manifestation of Parvovirus B19 Infection

**DOI:** 10.7759/cureus.43925

**Published:** 2023-08-22

**Authors:** Jun Tanimura, Ion Terakawa

**Affiliations:** 1 Department of Neurology, Aizawa Hospital, Matsumoto, JPN; 2 Department of General Internal Medicine, Aizawa Hospital, Matsumoto, JPN

**Keywords:** eyelid swelling, infectious mononucleosis-like syndrome, infectious mononucleosis, parvovirus b19, hoagland's sign

## Abstract

Parvovirus B19 infection typically exhibits a biphasic clinical course with varied symptoms. While facial or extremity edemas are common in adult cases, localized eyelid swelling, referred to as Hoagland's sign, had not been recognized as a potential clinical feature. Here, we present the case of a 72-year-old woman with parvovirus B19 infection and Hoagland's sign, characterized by drooping of the swollen upper eyelid and narrowing of the ocular aperture. The patient showed symptoms of fever, myalgia, and neutrophilia with atypical lymphocytes. The diagnosis was confirmed through elevated parvovirus B19 IgM antibody levels. This is the first reported case of parvovirus B19 infection presenting with Hoagland's sign. Parvovirus B19 infection can present with the condition called “infectious mononucleosis-like syndrome" (IML), which mimics classical manifestations of infectious mononucleosis (IM), including Hoagland's sign. Our case suggested that Hoagland's sign may be one of the characteristic symptoms of the second phase in the biphasic course of parvovirus B19 infection. It is important to consider the possibility of parvovirus B19 infection, especially in elderly patients presenting with Hoagland's sign.

## Introduction

Parvovirus B19 infection presents with highly variable symptoms along with its biphasic clinical course [1]. The initial viremic phase consists of nonspecific flu-like symptoms such as fever, malaise, myalgia, and headache, accompanied by hematologic abnormalities, such as anemia, leukopenia, and thrombocytopenia. Characteristic symptoms such as rash, arthralgia, and edema follow in a second phase that occurs approximately one week later [1]. In the clinical diagnosis of adult parvovirus B19 infection, it is critical to recognize the characteristic second-phase symptoms accurately. Although facial or extremity edemas are seen in parvovirus B19 infection [1], localized eyelid swelling with redness and mild pain, which is referred to as Hoagland’s sign in infectious mononucleosis (IM) or infectious mononucleosis-like syndrome (IML), had not been recognized as a potential clinical feature [2]. Here, we report a case of an elderly patient with parvovirus B19 infection presenting Hoagland’s sign. We aim to contribute to the early and accurate diagnosis of adult parvovirus B19 infection by highlighting its clinical diversity and sharing potential distinguishing signs.

## Case presentation

A 72-year-old woman presented with fever and eyelids swelling. Two weeks before the visit, she had a fever of up to 38 °C that lasted until the visit and generalized myalgia that lasted for a few days. A week before the visit, her eyelids began to swell, and, on the next day, the eyelid edema was so severe that she could hardly keep her eyes open. It was then partially resolved spontaneously. She had been in daily contact with her grandchildren; however, they did not suffer from any disease with fever, skin rash, or lymphadenopathy. Physical examination was unremarkable except for bilateral upper eyelids edema with redness, mild pain, and heat.

On the laboratory tests (Table 1), the white blood cell count in the peripheral blood was within the normal range, but marked neutrophilia (81%) with 2% atypical lymphocytes were recognized. Elevated C-reactive protein and ferritin levels demonstrated inflammation, and other biochemical findings were noted on elevated liver enzymes.

**Table 1:** Laboratory test findings.

Laboratory tests	Results	Reference
White blood cell (/mm^3^)	7,900	3,300–8,600
-Neutrophils (%)	81.0	45.0–72.0
-Lymphocytes (%)	10.0	20.0–45.0
-Atypical lymphocytes (%)	2.0	<1
Red blood cell (×10^4^/mm^3^)	385	386–492
Hemoglobin (g/dL)	12.6	11.6–14.8
Platelet (×10^4^/mm3)	33.3	15.3–34.8
Erythrocyte sedimentation rate (mm/hr)	90	<15
Prothrombin time-international normalized ratio	1.15	0.88–1.09
Aspartate aminotransferase (U/L)	38	13–30
Alanine aminotransferase (U/L)	42	7–23
Blood urea nitrogen (mg/dL)	7.0	8–20
Creatinine (mg/dL)	0.53	0.46–0.79
C-reactive protein (mg/dL)	12.42	<0.14
Ferritin (ng/mL)	504.5	12–60

The diagnosis of acute parvovirus B19 was confirmed due to elevated parvovirus B19 IgM antibody at a titer of 6.92 IU/mL (Table 2). IgM antibody titers of Epstein-Barr virus viral capsid antigen, Cytomegalovirus, and Toxoplasma gondii were negative. HIV antigen-antibody combination test was negative. The rheumatoid factor and antinuclear antibody titer were also negative.

**Table 2:** Infectious and immunological testing. ANCA: Anti-neutrophil cytoplasmic antibody, CMV: Cytomegalovirus, EA: Early antigen, EBNA: Epstein-Barr nuclear antigen, EBV: Epstein-Barr virus, HIV: Human immunodeficiency virus, VCA: Viral capsid antigen.

Laboratory tests	Results	Reference	Interpretation
EB-VCA IgG (IU/mL)	9.9	<0.5	+
EB-VCA IgM (IU/mL)	0	<0.5	−
EB-EA IgG (IU/mL)	0.8	<0.5	±
EBNA (IU/mL)	40	<10	+
CMV IgG (IU/mL)	2	<2.0	−
CMV IgM (IU/mL)	0.24	<0.8	−
Toxoplasma gondii IgG (IU/mL)	112	<6	+
Toxoplasma gondii IgM (IU/mL)	0.2	<0.8	−
Parvovirus B19 IgM (IU/mL)	6.92	<0.8	+
Hepatitis B surface antigen (IU/mL)	0.040	<0.049	−
Hepatitis B surface antibody (mIU/mL)	0.5	<10.0	−
Hepatitis C antibody (signal-to-cutoff ratio)	0.04	<0.99	−
HIV antigen/antibody	Negative	Negative	−
Rheumatoid factor (IU/mL)	2.9	<15	−
Antinuclear antibody	<40	<40	−
MPO-ANCA (U/mL)	<1.0	<3.5	−
PR3-ANCA (U/mL)	<1.0	<3.5	−

The patient received supportive treatment, and symptoms and laboratory abnormalities resolved a week after the visit.

## Discussion

To our knowledge, this is the first report of a parvovirus B19 infection with Hoagland’s sign, characterized by drooping of the swollen upper eyelid and narrowing of the ocular aperture [3].

Acute parvovirus B19 infection can sometimes present as an infectious mononucleosis-like syndrome (IML) [2,4], which exhibits some of the classical manifestations of infectious mononucleosis (IM), such as fever, pharyngitis, and lymphadenopathy with the presence of atypical lymphocytosis [5]. IML has been reported to be associated with Epstein-Barr virus (EBV), cytomegalovirus (CMV), human herpesvirus 6 (HHV-6), human immunodeficiency virus (HIV), adenovirus, herpes simplex virus (HSV), parvovirus B19, Streptococcus pyogenes and Toxoplasma gondii [4,5], with all of these forming a cluster of diseases with mimetic presentations. Although Hoagland’s sign is one of the classical manifestations of IM [3,6,7], reports of IML with Hoagland’s sign have been limited to cases of Cytomegalovirus [8], and it has never been reported in patients with parvovirus B19 infection. Of note, unspecified facial edema was reported in 14.3% of patients with acute parvovirus B19 infection, which appeared as the second-phase symptom in all patients [1]. It is possible that these may have included cases localized to the eyelids, which could be termed Hoagland's sign.

Our case suggests that Hoagland's sign may present as a second-phase symptom of acute parvovirus B19 infection. It is often difficult to consider the possibility of parvovirus B19 infection unless the characteristic second-phase symptoms appear, as the symptoms of the initial phase are nonspecific. However, even in the second phase, only 67.9% of cases present with both rash and arthralgia [1]. Noteworthy, edema, which has not received much attention [9,10], was observed in a non-negligible number of cases (43.3-92.9%) [1,11]. Therefore, it is important to rediscover the diagnostic value of edema, including Hoagland's sign, to improve the diagnostic accuracy of parvovirus B19 infection.

In our diagnostic process, we first recalled IM and the cluster of IML by Hoagland's sign, which led to the diagnosis (the pivot and cluster strategy [12]). Since >95% of EBV infection occurs within the first decades of life [13], elderly-onset IML suggested other pathogens, including acute parvovirus B19 infection, which occurs between the ages of 25 and 65 [1]. Taken together with this epidemiologic information on IML, it may be important to consider parvovirus B19 infection when an elderly patient presents with Hoagland's sign.

## Conclusions

Hoagland's sign may be one of the characteristic symptoms of parvovirus B19 infection, in the second phase of its biphasic course. When considering parvovirus infection, sick contact, biphasic course, and edema, including Hoagland's sign, in addition to rash and arthralgia, should be examined. Conversely, in patients with Hoagland's sign, the possibility of acute parvovirus B19 infection should be considered.
